# Elimination of lipaemic interference by high-speed centrifugation

**DOI:** 10.11613/BM.2023.010703

**Published:** 2022-12-15

**Authors:** Gemma Solé-Enrech, Ruth Cano-Corres, Maria Isabel Aparicio-Calvente, Nino Spataro

**Affiliations:** Clinical laboratory, Biochemistry Department, Parc Taulí Research and Innovation Institute Foundation (I3PT), Sabadell, Spain

**Keywords:** lipaemia, high-speed centrifugation, lipaemic index

## Abstract

**Introduction:**

In order to deliver high quality results, detection and elimination of possible analytical interferences, such as lipaemia, is crucial. The aim of this study is to evaluate the efficacy of high-speed centrifugation in eliminating lipaemic interference and to define own lipaemic index (LI) for the studied biochemical analytes.

**Materials and methods:**

Evaluated analytes were: albumin, alkaline phosphatase, alanine-aminotransferase (ALT), aspartate-aminotransferase (AST), calcium, creatinine, gamma-glutamyltransferase (GGT), glucose, phosphates, total proteins, urea and total bilirubin. Those analytes and LIs have been analysed in duplicate in the Roche Diagnostics-c8000 analyser in samples centrifuged at 3000 rpm/10 minutes in the SL16 (Thermo Scientific, Waltham, USA) centrifuge and according to an own high-speed centrifugation protocol (12,900 rpm/15 minutes) in the MicroCL17R (Thermo Scientific, Waltham, USA) centrifuge. Lipaemia has been measured in each sample. The efficiency of high-speed centrifugation is verified by the Wilcoxon test (P < 0.05). In cases where significant differences are observed, our own LI is calculated. For ALT and AST, it is verified by McNemar test (P < 0.05*)*. For creatinine, both Wilcoxon and McNemar test were applied.

**Results:**

There were statistically significant differences in analyte concentration before and after high-speed centrifugation for: albumin, creatinine, GGT, glucose, phosphates, urea and total bilirrubin. Own LI is calculated. McNemar test shows statistically significant diferences in the proportion of delivered results before and after high-speed centrifugation in ALT, AST and creatinine.

**Conclusions:**

This study confirms the efficacy of high-speed centrifugation protocol for all the considered analytes, excepting calcium, alkaline phosphatase and total proteins.

## Introduction

Analytical interferences, such as lipaemia, haemolysis and icteria, if not detected can affect the validity of results delivered by clinical laboratory. Lipaemia is one of the most frequent analytical interferences in clinical laboratory and it is due to a high concentration of fats, producing a naked eye detectable turbidity in serum or plasma.

Lipaemia can affect the measurement of biochemical analytes in different ways. Since lipid particles are able to absorb light, they can interfere in spectrophotometric methods, especially with those using lower wavelengths.

In addition, lipaemia interfere with electrophoretic methods, modifying the morphology of the alpha-2-globulin fraction, and with methods that measure analytes in total plasma volume, such as indirect potentiometry decreasing electrolytes concentration. Finally, lipids particles are also known to interfere in immunoassays, blocking antibodies binding sites (*1*).

Until few years ago, presence of lipaemia was assessed by the naked eye, a laborious and subjective method. Nowadays, analysers incorporate systems to detect interferences, known as warning index of haemolysis, icteria and turbidity. These systems are based on the spectral characteristics of haemoglobin, bilirubin and turbidity and enable the automatic detection of interferences, increasing objectivity, sensibility and speed. Warning indexes are calculated taking into account absorbance values obtained in the sample at a certain wavelength (*2*). Focusing on lipaemia, if the analyser detects the presence of turbidity, the next step is to remove it. This way, the laboratory ensures the delivery of real results.

According to Clinical and Laboratory Standards Institute (CLSI), the gold standard method to eliminate lipaemic interference is ultracentrifugation (*3*). Ultracentrifuges obtain forces up to 100,000 rpm allowing the effective removal of lipids. Since, ultracentrifuges are not available in many laboratories, commerical kits, such as Lipoclear, or organic polar solvents, such as polyethylene glycol, n-hexane or cyclodextrin, have been developed and used as an alternative to ultracentrifuge to remove lipids from the sample. Organic solvents are able to bind to the lipid particles due to their precipitation and allow to obtain a valid measurement in the supernatant (*1*).

However, these solvents do not eliminate the whole lipaemic interference; in addition, they can interfere in the measurement of some biochemical analytes (*4*, *5*). For this reason, some authors recommend the use of high-speed centrifugation as a valid alternative (*4*-*6*).

It is important for each laboratory to verify the employed lipaemic index (LI), because differences could be observed comparing with those provided by the manufacturer. This information would allow each laboratory to decide how restrictive wants to be, and not automatically accept manufacturers’ LIs.

The aim of this study is to evaluate the efficacy of high-speed centrifugation in eliminating lipaemic interference and to define a custom LI for the studied biochemical analytes.

## Material and methods

### Materials

From May 2020 to January 2022, a total number of 174 serum samples have been processed in a prospective study. The research protocol has been developed in a clinical laboratory certified according to ISO 9001:2015.

Samples are drawn in gel separator tubes without anticoagulant (Vacuette Z serum sep clot activator, Greiner Bio-One, Kremsmünster, Austria). Samples used in this study are obtained from out-patients referred to the laboratory for blood testing and from in-patients. After laboratory testing is done, remaining serum samples are used for the study purposes. No additional blood sampling was needed for this research.

The following biochemical analytes have been evaluated in this project: albumin, alkaline phosphatase, alanine aminotransferase (ALT), aspartate aminotransferase (AST), calcium, creatinine, gamma-glutamyltransferase (GGT), glucose, phosphates, total proteins, urea and total bilirubin. Since not all the considered analytes were measured in the whole set of 174 serum samples, a specific number of measurements was obtained for each specific analyte.

The study has been approved by the Hospital’s ethics committee of clinical research (CEIM code:2020672*).*

### Methods

All analytes have first been measured in centrifugated serum samples according to the protocol of centrifugation of serum samples established by the laboratory which indicates the centrifugation of samples at 3000 rpm for 10 minutes. The centrifuge used was SL16 (Thermo Scientific, Waltham, USA).

Then, the same serum has been centrifugated at high-speed at 12,900 rpm for 15 minutes and the same biochemical analytes have been measured again. The centrifuge used for high-speed centrifugation was MicroCL 17R (Thermo Scientific, Waltham, USA).

Analytes measurements have been obtained using a Cobas 8000 automated analyser (Roche Diagnostics, Mannheim, Germany). The specific methods used for each analyte are shown in Table 1.

**Table 1:** Overview of methods used in the measurement of the biochemical analytes in the study

**Biochemical magnitudes**	**Methods**	**LI (Roche)**
Albumin	Colorimetric method using bromocresol green	500
Alkaline phosphatase	Colorimetric method using para-Nitrophenylphosphate (AMP buffer)	2000
Calcium	BAPTA (Bisaminophenox tetraacetic acid) and EDTA	1000
Creatinine	Colorimetric compensated test based on Jaffé method	800
GGT	G-glutamyl-carboxy-nitroanilide - IFCC Ref. Proc., Calibrated	700
Glucose	UV Enzymatic method using hexokinase	1000
Phosphates	UV Phosphomolybdat	1250
Total proteins	Colorimetric test. Purpureo Biuret	2000
Urea	Kinetic test with urease and glutamate dehidrogenase UV	1000
Total bilirrubin	Diazo colorimetric method	1000
ALT	UV without pyridoxal phosphate activation	150
AST	UV without pyridoxal phosphate activation	150
LI - the highest acceptable lipaemic index proposed by Roche (Roche Diagnostics, Mannheim, Germany) for each parameter. GGT - gamma-glutamyltransferase. ALT - alanine aminotransferase. AST - aspartate aminotransferase. UV – ultraviolet.		

### Statistical analysis

Data distribution has been analysed using Kolmogorov-Smirnov test (P < 0.05). Statistically significant differences between centrifugation protocols have been assessed using a Wilcoxon test. When significant differences were observed, a custom LI was calculated and compared to the one established by the manufacturer.

For each analyte, the calculation of the custom LI is done in the following way. First of all, the percentage difference between the results obtained before and after high-speed centrifugation is determined. This percentage difference has been calculated using the concentration of analyte after high-speed centrifugation as denominator. Then, a linear regression analysis is performed using the value of lipaemia obtained in the primary tube (centrifugation according to the usual protocol) and the percentage difference. In order to establish the value of LI over which the studied interference is considered significant, we selected the maximum permissible error (MPE) in the quality specification working paper of the Spanish Society of laboratory medicine (SEQC-ML) (*7*). The value of LI from which the MPE is exceeded is calculated with the equation of the linear regression and the selected MPE.

Due to lipaemic interference and the warnings derived, some of the analytes couldn’t be delivered by the laboratory. McNemar test is used to compare the number of results provided by the laboratory before and after high-speed centrifugation. This procedure was carried out considering all samples of the study. In a separate test, only samples with lipaemia levels > 150 were considered, since according to the manufacturer this is the lowest lipaemic index affecting the analytes included in the study (see Table 1).

The statystical analysis was performed using the statistical software Analyse-it for Microsoft Excel (version 5.9) (Microsoft, Leeds, United Kindom).

## Results

Lipaemic index obtained in the samples analysed before high-speed centrifugation ranged from 1 to 2699 (median: 113; interquartile range (IQR): 27-249.5).

Statistically significant differences between results obtained from standard and high-speed centrifugation were observed for the following biochemical analytes: albumin, creatinine, GGT, glucose, phosphates, urea and total bilirubin (Table 2). For these analytes, the LI is calculated following the protocol explained in the previous section. On the contrary, no differences were observed for alkaline phosphatase, calcium and total proteins, so LI have not been calculated.

**Table 2:** Results of the studies for LIs definition

**Biochemical analytes (unit)**	**N**	**P value (Wilcoxon)**	**Before high-speed centrifugation** **median (IQR)**	**After high-speed centrifugation** **median (IQR)**	**Linear regression equation**	**MPE** **(%)**	**LI** **(Roche)**	**LI (study)**
Albumin (g/L)	113	P < 0.001	40.3 (33.7-45.4)	40.9 (35-46.3)	% DIF = 0.99 + 0.01 LI	5.2	550	593
Alkaline phosphatase (U/L)	127	P = 0.112	89 (73-139)	89 (72-140)	-	10.6	2000	-
Calcium (mmol/L)	86	P = 0.498	2.32 (2.17-2.44)	2.34 (2.17-2.44)	-	3.8	1000	-
Creatinine (µmol/L)	144	P < 0.001	78.32 (56.55-100.18)	84.07 (68.94-102.92)	% DIF = 5.21 + 0.02 LI	7.4	800	145
GGT (U/L)	126	P < 0.001	41 (21-134)	40 (21-136)	% DIF = - 0.56 + 0.02 LI	8.8	700	612
Glucose (mmol/L)	143	P < 0.001	6.72 (5.22-13.49)	6.66 (5.33-13.66)	% DIF = - 0.09 + 0.01 LI	6.4	1000	1017
Phosphates (mmol/L)	90	P < 0.001	1.16 (1-1.32)	1.19 (1-1.32)	% DIF = 0.77 + 0.01 LI	5.1	1250	511
Total proteins (g/L)	96	P = 0.268	0.68 (0.58-0.73)	0.69 (0.59-0.73)	% DIF = - 0.34 + 0.01 LI	3.5	2000	-
Urea (mmol/L)	144	P < 0.001	5.79 (4.50-7.58)	5.81 (4.61-7.53)	% DIF = - 0.94 + 0.01 LI	8.9	1000	698
Total bilirrubin (µmol/L)	111	P < 0.001	6.15 (3.76-10.26)	5.98 (3.59-9.91)	% DIF = - 9.64 + 0.01 LI	13.5	1000	1691
LI - lipaemic index. GGT - gamma-glutamyltransferase. MPE - maximum permissible error. IQR – interquartile range. Regression - % DIF is the percentage difference of the results before and after high-speed centrifugation, LI is the lipaemic index measured by Roche Cobas 8000 analyser in samples centrifuged by conventional protocol.								

**Figure 1:**
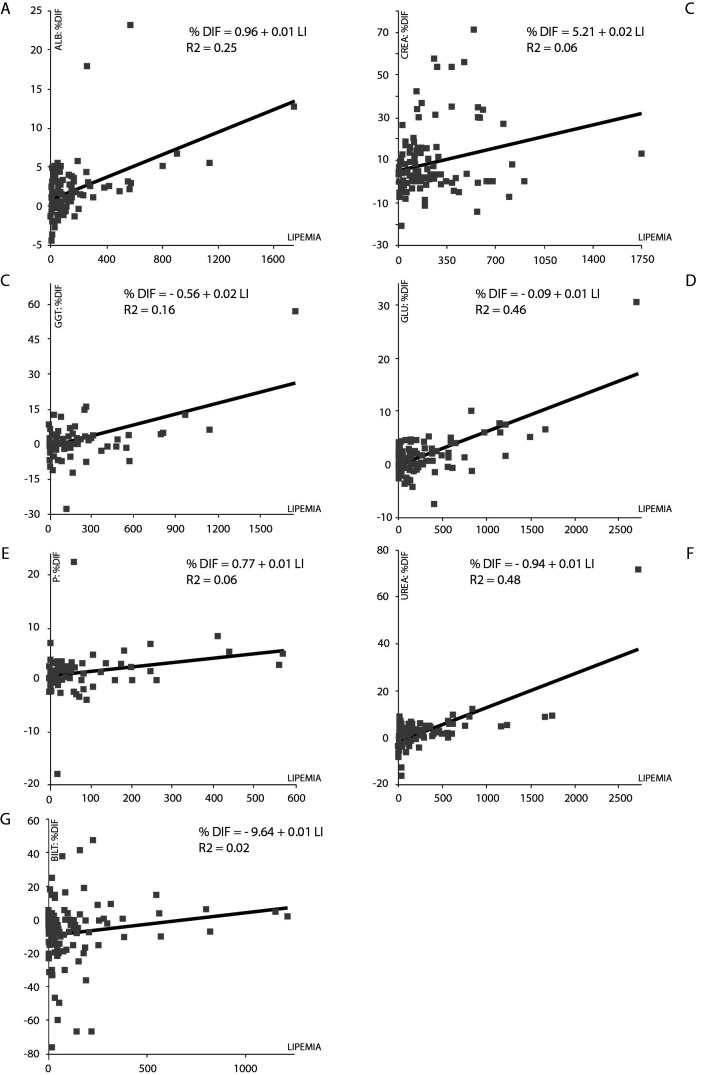
Linear regression graphics. A) Albumin; B) Creatinine; C) Gamma-glutamyltransferase (GGT); D) Glucose; E) Phosphate; F) Urea; G) Total bilirubin.

Descriptive statistics, the equation of the linear regression analysis, and MPE used for custom LIs calculation and the differences between the LIs proposed by the manufacturer and the LIs obtained in this study are shown in Table 2. A graphical representation of the linear regression analysis is available in Figure 1.

In order to test if the proportion of results delivered by the analyser without lipaemia warning has increased after the high-speed centrifugation, a McNemar test was carried out for the analysis of creatinine, ALT and AST. Using the conventional protocol 89%, 87% and 91% of creatinine, ALT and AST results, respectively, did not show lipaemia warning. After high-speed centrifugation 99%, 97% and 97% of creatinine, ALT and AST results, respectively, did not present lipaemia warning and could be delivered to the physician (Table 3). Considering the subset of samples with LI > 150, the percentages of results delivered to the physician significantly increased from 80% to 100% for creatinine, from 67% to 96% for ALT and from 76% to 96% for AST when passing from the usual protocol to high-speed centrifugation (Table 3). In all cases the differences have been statistically significant.

**Table 3:** Comparison of results delivered before and after high-speed centrifugation

**Biochemical analytes**	**N**	**Results delivered before high-speed centrifugation N (%)**	**Results delivered after high-speed centrifugation** **N (%)**	**P**
Creatinine	164*	146 (89)	163 (99)	< 0.001
	64^†^	51 (80)	64 (100)	< 0.001
ALT	149*	129 (87)	145 (97)	< 0.001
	49^†^	33 (67)	47 (96)	< 0.001
AST	151*	138 (91)	147 (97)	0.013
	49^†^	38 (76)	47 (96)	0.003
*All samples. ^†^Samples with LIs > 150. LI – lipaemic index. ALT - alanine aminotransferase. AST - aspartate aminotransferase.				

## Discussion

The current study demonstrates the usefulness of high-speed centrifugation for the removal of lipaemia interference. For most of the considered analytes, high-speed centrifugation increases significantly the percentage of valid results delivered by our laboratory. Taking advantage of the data generated in our study, custom LI values have been obtained for the analytes for which significant differences were observed between the usual protocol and high-speed centrifugation.

Several studies indicate that the gold standard method for the removal of lipaemic interference is ultracentrifugation, which is a very expensive method and therefore not available for all laboratories. Some authors have evaluated the efficacy of high-speed centrifugation as well as the use of organic solvents to remove lipids from the samples (*4*, *5*).

This study confirms that high-speed centrifugation can potentially improve the management of lipaemic samples in the laboratory.

Custom LI were not obtained only for alkaline phosphatase, calcium and proteins, for which no differences were observed between the tested protocols. Given the high LI suggested by the manufacturer for the usual protocol, we believe that the analytical methods used to measure the concentration of these three analytes are not influenced by lipaemia interference (Table 1).

In the case of creatinine, the custom LI is much lower than the one suggested by the manufacturer (145 *vs*. 800). This result could explain the elevated number of undetectable results (< 15 µmol/L) observed in patients not suffering from renal illness. In this study, 100% of the undetectable creatinine results have turned detectable, showing values within the reference interval after high-speed centrifugation. Similarly to what reported from Nikolac *et al.,* for creatinine we observed a discrepancy between the influence of lipaemia declared by the manufacturer and the one obtained experimentally, suggesting that lipaemic interference was underestimated by the manufacturer (*8*). It is important to remark that this study has been made with the creatinine measured by Jaffé compensated method, and nowadays the recommended method to measure creatinine is the enzymatic method. Laboratories employing enzymatic methods should verify their own LIs.

Likewise, for phosphates, GGT and urea, the LIs obtained in this study have also been more restrictive than the ones proposed by the manufacturer. On the contrary, for total bilirubin, the LI provided by the manufacturer is more restrictive. Nikolac *et al.* also reported overestimation of lipaemia interference for total bilirubin when investigating the manufacturer’s LI for Roche reagents (*8*). We believe that the higher MPE chosen for custom LI calculation compared to the MPE selected by the manufacturer, which is usually about 10%, could explain the observed differences (Table 2). Finally, for albumin and glucose, custom LIs and those suggested by manufacturer were similar.

Interestingly, the results obtained from the analysis of ALT, AST and creatinine demonstrates that the application of high-speed centrifugation to eliminate the lipaemic interference allows the laboratory to deliver results that couldn’t have been delivered otherwise, which in turn improves analytical quality and patient care.

One of the main limitations of this study is the impossibility of calculating the LI for ALT and AST. Since for those analytes the interference was so high, many of the results delivered by the analyser produced lipaemic warnings. Moreover, even if haemolysis index was measured, we did not investigate if it could affect our results. Since, the haemolysis index was similar before and after high-speed centrifugation, we suppose that haemolysis index should not affect our results. In addition, high-speed centrifugation is not described in literature to remove haemolysis interference, further suggesting that hemolysis should not affect our results.

This article makes a difference calculating its own LI. In addittion, the LIs have been calculated considering the MPE, which is a tougher and more adequate criterion than the one used by the manufacturer, which is about 10%.

The evidences provided by our study indicate that each laboratory should calculate its custom LIs and compare them to those proposed by the manufacturer in order to assure high quality of the delivered results.

The definition of own LIs would improve quality results thus beneficiating patient care, especially for the analytes whose results frequently can’t be delivered due to warning alarms.

In conclusion, this present study confirms the efficacy of high-speed centrifugation protocol for all the considered analytes, except for calcium, alkaline phosphatase and total proteins.
